# Privacy-Preserving Federated Model Predicting Bipolar Transition in Patients With Depression: Prediction Model Development Study

**DOI:** 10.2196/46165

**Published:** 2023-07-20

**Authors:** Dong Yun Lee, Byungjin Choi, Chungsoo Kim, Egill Fridgeirsson, Jenna Reps, Myoungsuk Kim, Jihyeong Kim, Jae-Won Jang, Sang Youl Rhee, Won-Woo Seo, Seunghoon Lee, Sang Joon Son, Rae Woong Park

**Affiliations:** 1 Department of Biomedical Informatics Ajou University School of Medicine Suwon-si Republic of Korea; 2 Department of Biomedical Sciences Ajou University Graduate School of Medicine Suwon-si Republic of Korea; 3 Department of Medical Informatics Erasmus University Medical Center Rotterdam Netherlands; 4 Observational Health Data Analytics Janssen Research and Development Titusville, NJ United States; 5 Data Solution Team Evidnet Co, Ltd Sungnam Republic of Korea; 6 Department of Neurology Kangwon National University Hospital Kangwon National University School of Medicine Chuncheon Republic of Korea; 7 Center for Digital Health Medical Science Research Institute Kyung Hee University Medical Center Seoul Republic of Korea; 8 Department of Endocrinology and Metabolism Kyung Hee University College of Medicine Seoul Republic of Korea; 9 Department of Internal Medicine Kangdong Sacred Heart Hospital Hallym University College of Medicine Seoul Republic of Korea; 10 Department of Psychiatry Myongji Hospital Goyang Republic of Korea; 11 Department of Psychiatry Ajou University School of Medicine Suwon-si Republic of Korea

**Keywords:** federated learning, depression, bipolar disorder, data standardization, differential privacy

## Abstract

**Background:**

Mood disorder has emerged as a serious concern for public health; in particular, bipolar disorder has a less favorable prognosis than depression. Although prompt recognition of depression conversion to bipolar disorder is needed, early prediction is challenging due to overlapping symptoms. Recently, there have been attempts to develop a prediction model by using federated learning. Federated learning in medical fields is a method for training multi-institutional machine learning models without patient-level data sharing.

**Objective:**

This study aims to develop and validate a federated, differentially private multi-institutional bipolar transition prediction model.

**Methods:**

This retrospective study enrolled patients diagnosed with the first depressive episode at 5 tertiary hospitals in South Korea. We developed models for predicting bipolar transition by using data from 17,631 patients in 4 institutions. Further, we used data from 4541 patients for external validation from 1 institution. We created standardized pipelines to extract large-scale clinical features from the 4 institutions without any code modification. Moreover, we performed feature selection in a federated environment for computational efficiency and applied differential privacy to gradient updates. Finally, we compared the federated and the 4 local models developed with each hospital's data on internal and external validation data sets.

**Results:**

In the internal data set, 279 out of 17,631 patients showed bipolar disorder transition. In the external data set, 39 out of 4541 patients showed bipolar disorder transition. The average performance of the federated model in the internal test (area under the curve [AUC] 0.726) and external validation (AUC 0.719) data sets was higher than that of the other locally developed models (AUC 0.642-0.707 and AUC 0.642-0.699, respectively). In the federated model, classifications were driven by several predictors such as the Charlson index (low scores were associated with bipolar transition, which may be due to younger age), severe depression, anxiolytics, young age, and visiting months (the bipolar transition was associated with seasonality, especially during the spring and summer months).

**Conclusions:**

We developed and validated a differentially private federated model by using distributed multi-institutional psychiatric data with standardized pipelines in a real-world environment. The federated model performed better than models using local data only.

## Introduction

### Background

Mood disorders such as depression and bipolar disorder (BD) significantly cause disability that severely limits individual psychosocial functions and lowers the quality of life [1]. BD has a less favorable prognosis and needs treatments different from those needed for depression, although both have overlapping symptoms and pathophysiology [2,3]. Prompt recognition of depression conversion to BD may help prevent the negative consequences of BD because BD is initially confused with depression [4,5]. However, early BD recognition or prediction among people with depression is challenging due to delayed diagnostic differentiation.

Several machine learning studies have been conducted to predict the diagnostic transition from depression to BD. One study [6] developed models with an area under the curve (AUC) of 0.76. However, the model was developed using a single center, and the performance deteriorated to ≤0.71 in external validation. Nestsiarovich et al [7] developed a prediction model of the conversion from depression to BD across multiple databases in the United States. That model had poor performance during external validation and its overall performance was modest. Further, that study [7] was conducted using data centralization, which cannot be universally adopted in many nonprofit institutions due to privacy and regulatory concerns [8], especially in highly sensitive fields such as psychiatry [9].

Recently, McMahan et al [10] proposed a novel federated learning (FL) algorithm that trains a universal model across several clients without data centralization. Global weights are initially distributed to individual clients in each round of FL, and the model is trained in the local data set. Then, the weights are updated with the local data set and transmitted to the global server and aggregated, and this round is repeated for a predetermined number of rounds. FL is considered a privacy-preserving method for training a multi-institutional model by sharing only model weights and not patient-level data. Some medical studies using FL, such as those predicting clinical outcomes in patients with COVID-19 [11], revealed the feasibility of FL. However, the following obstacles remain in medical FL: (1) absence of a standardized data pipeline, (2) privacy leakage, and (3) hardware limitations [12].

The first challenge is the absence of a standardized feature extraction pipeline. Each hospital has a different electronic health record structure. Local research collaborators have to preprocess and extract features themselves without a standardized pipeline. Hence, individual analysis codes are generated in as many as the number of hospitals, thereby reducing transparency, reproducibility, and interoperability and causing data quality instability that adversely affects the overall model performance [13]. This process consumes enormous resources for each hospital, thereby slowing the research progress, reducing scalability, and preventing data-driven approaches. Therefore, conducting FL on a standardized feature extraction pipeline that works universally across these hospitals is required.

The second challenge is privacy leakage, which can occur during both feature extraction and model development. A person with prior information is at risk of identifying the patient in feature extraction, even with anonymized data [14]. In particular, the risk of such patient identification is higher in small hospitals, suggesting that patient-level data access should be minimized even for local researchers. The shared weights may indirectly expose patient-level data in model development. Moreover, the weight sharing is repeated in several rounds, thereby increasing the risk of leakage. Adequate countermeasures such as differential privacy to quantify and limit privacy leakages are required [13,15].

The third challenge is hardware limitations. Most hospitals lack the computational resources for FL. The training speed of FL is tailored for the slowest client because the weights of all hospitals must be gathered before proceeding. The FL process can exclude some hospitals with a slow training speed, thereby reducing the available data and making the model less robust. Medical data are well-known for sparsity, and the model training time is generally known to be reduced while maintaining the model performance through appropriate feature selection [16]. Thus, overcoming the hardware limitation by finding a way to apply feature selection in an FL environment is necessary.

### Objectives

In this study, we aimed to develop a federated bipolar transition prediction model that overcomes the obstacles of FL. This study aims to standardize the feature extraction pipeline, apply differential privacy, reduce the model training time, and develop a standardized single analysis code for all hospitals.

## Methods

### Data Sources

We recruited 4 teaching hospitals, namely, Ajou University School Of Medicine (AUSOM; January 1994 to February 2022), Kyung Hee University Medical Center (KHMC; January 2008 to February 2022), Myongji Hospital (MJ; June 2006 to October 2021), and Kangdong Sacred Heart Hospital (KDH; January 2005 to October 2021) to develop the model. We used Kangwon National University Hospital (KW; January 2003 to January 2022) located in a geographically administratively separate area for external validation of the model. All development and external validation participating hospitals were teaching hospitals from other universities and foundations. We used Observational Medical Outcomes Partnership Common Data Model (OMOP CDM) as the data format. OMOP CDM is a set of uniform data standards that regulates the format and content of observational data maintained by the Observational Health Data Sciences and Informatics (OHDSI) [17,18]. OHDSI is an interdisciplinary collaboration undertaken by a multi-stakeholder group to discover the value of observational data. More than 2000 researchers worldwide participate in OHDSI, and 74 countries have converted or are converting data to OMOP CDM [19]. The electronic medical record data in South Korea from 37 hospitals with 50 million patients were converted to OMOP CDM [20]. Then, the data-converted hospitals formed a network called FeederNet (Federated E-Health Big Data for Evidence Renovation Network), a bio-health big data platform that was invented by RWP and supported by the Korean National Project to collaborate with OHDSI networks. The electronic medical record patient data of each hospital were pseudonymized and standardized based on OMOP CDM and stored in each institution. Each hospital’s extract, transform, and load process to OMOP CDM was conducted by an honest broker in the hospital; thus, we used only data from which personal information was already deleted. Data quality was checked using the automated Achilles tool and regularly inspected by experts [21].

### Overview of Model Development

**Figure 1:**
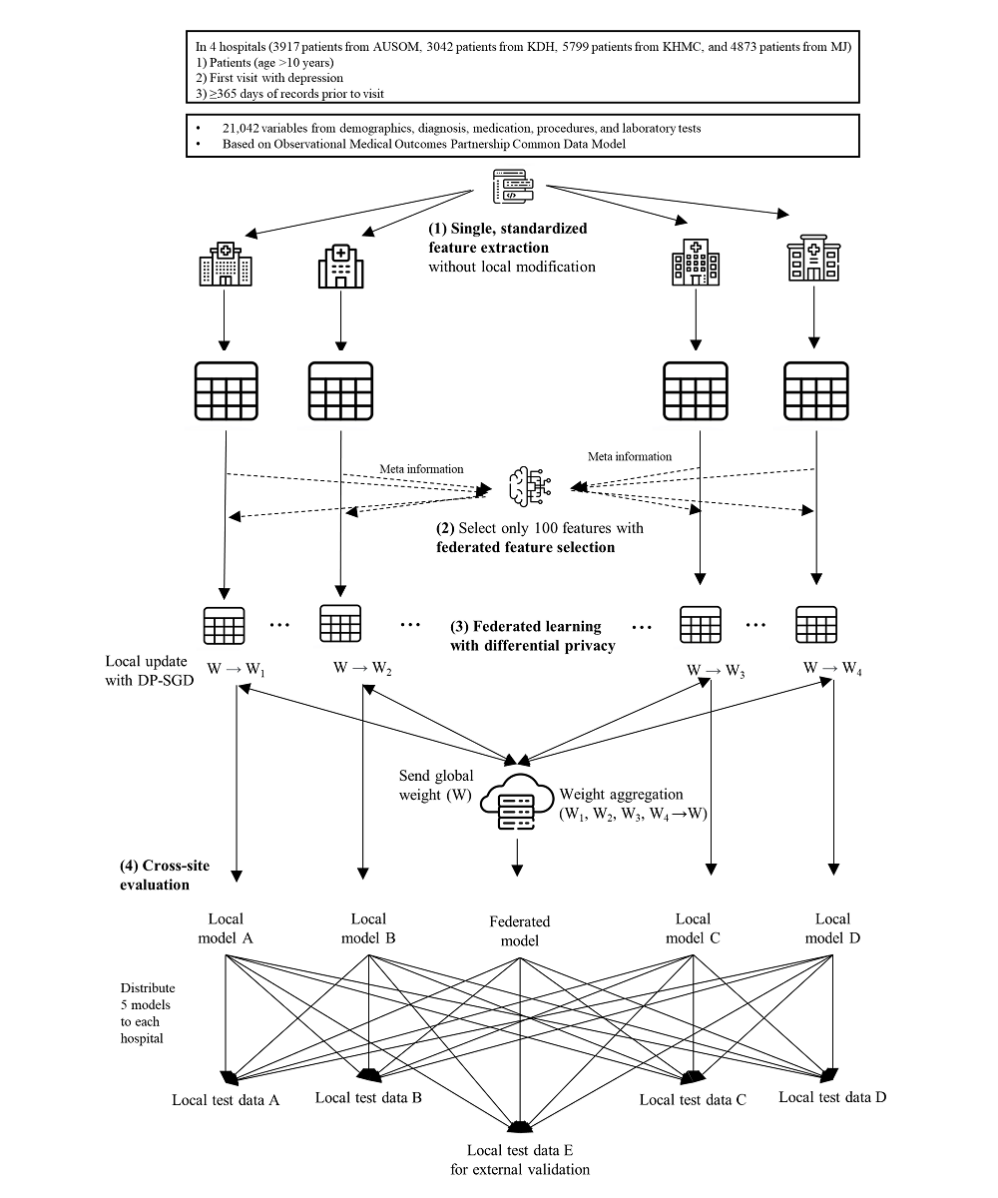
Overall workflow of the study. AUSOM: Ajou University School Of Medicine; DP-SGD: differential private-stochastic gradient descent; KDH: Kangdong Sacred Heart Hospital; KHMC: Kyung Hee Medical Center; MJ: Myongji Hospital.

This study model was developed and validated through 4 stages: (1) standardized feature extraction, (2) federated feature selection, (3) FL, and (4) cross-site evaluation. Figure 1 illustrates an overview of the model development. In the first step, we extracted features from all research-participating hospitals with the standardized feature extraction code. We reduced the number of features while maintaining model performance in the federated feature selection. Then, we conducted FL. The FL process entailed sending the global weight from the central server to the client, performing a local update on each client, and aggregating the weights. The above iteration was repeated. We used the federated averaging algorithm for the weight aggregation algorithm and applied differential private-stochastic gradient descent (DP-SGD) during the local update to ensure differential privacy. Through these processes, we successfully developed a federated, differentially private deep learning model based on the selected features without sharing patient-level data. In addition, we developed a deep learning model using only each client’s local data to compare model performance. After model development, we validated the model by using cross-site evaluation. We compared the performance of the federated model with the local models and performed external validation at a hospital that did not participate in the model development.

### Study Participants

Previously validated cohort definitions and extraction codes from Nestsiarovich et al [7] were used with the OMOP CDM. The target cohort was defined as individuals with the first observable diagnosis of depression, and the outcome was defined as having a BD diagnosis within 1 year after the depression diagnosis. The study population included patients with depression. The index date was defined as the patient’s first depression diagnosis record. The inclusion criteria are age >10 years at the index date; at least 1 year of observation before the index date; no BD, schizophrenia, or schizoaffective disorder diagnosis before the index date; and no neuroleptics (antipsychotics, antidepressants, mood stabilizers, and anticonvulsants) before the index date. All participants were followed up for 1 year or up to the day of BD diagnosis coding. The features included patient demographics, sex, age, diagnoses, drug exposures, procedures, and measurements. More than 20,000 features were included. The study code was sent to each client without modification, and the data were extracted from the client OMOP CDM database and saved to the client training server.

### Standardized Feature Extraction

The features used in the model were standardized according to the OMOP CDM. Study cohorts and outcome populations were defined using standardized features. Cohort and feature definitions produced by OHDSI ATLAS are printed in JSON format. ATLAS is an interactive analysis platform for OHDSI analysis tools [22]. We extracted labeled features by connecting the JSON configuration to the CDM database and storing the features in each client’s local server for FL. We also publicly share the JSON configurations on GitHub for transparency and reproducibility [23].

### Federated Feature Selection

One of the major challenges with FL is the lack of computational resources. This study trained the model by using only the central processing unit (CPU) in all the hospitals. Thus, local training in each hospital was the main bottleneck in our case rather than the network communication problem. We had to distill the features through federated feature selection to accelerate model development.

In the above process, we had to search for the number of input features (optimal N) because the learning speed will be slow if N is too large and performance will be too poor if N is too small. However, investigating all search spaces of N is extremely expensive. Accordingly, we set the initial N to 25 and performed FL to calculate the weighted mean AUC in the validation data set of 4 hospitals. Then, we increased N, trained the federated model, and calculated model performance. We repeated the above search, continuously increasing N. However, we stopped the optimization early if the model performance did not increase by more than 2% in 3 consecutive searches. Specifically, we trained a local gradient boosting model on each client’s training data set and aggregated the feature gain importance of each local boosting model and averaged out feature importance. We used the top N features for FL based on averaged feature importance. Moreover, we only included features that were present in all of the internal data sets. We chose the LightGBM algorithm because it quickly trains even on CPU and has repeatedly proven its feasibility on medical tabular data sets [24]. Every hyperparameter uses the default value of the Python LightGBM package.

### FL Process

We used FL, which was proposed by McMahan et al [10], for the development of a multicenter model without patient-level data sharing. The following 4 steps were repeated for each round in FL: (1) the global model weights are sent to each client, (2) each client trains the model on its data set, (3) only locally updated model weights and related meta information are sent to the central server, and (4) the new global model weights are computed by aggregating the centrally transmitted local model updates. The total rounds and the epochs of local training are hyperparameters. Our study trained only 5 rounds in FL and allowed 5 epochs to be trained in each round. We used federated averaging as the aggregation step [10]. The loss was aggregated from each client’s local tuning set in every round of FL. The model of the round with the lowest aggregated loss was used as the final model.

We used Deep and Cross Network (DCN) as the model algorithm [25], which showed feasibility in medical FL [11,25]. DCN is a model suitable for sparse large tabular data sets and has been used in medical FL. It utilizes data embedding, stacking, and cross-networking while maintaining deep neural network strength. We developed a local model based on the DCN algorithm using only each client’s training and tuning data set to compare with the federated model. We trained the model for 25 epochs in each client. The model of the epoch with the lowest loss was selected as the final local model.

### Application of Differential Privacy

Additionally, we used DP-SGD in the model training to apply differential privacy in model development [15]. Differential privacy is a strong privacy guarantee for FL. Differential privacy quantifies privacy loss generated in data use. It allows us to compare and control the privacy loss of different settings. Pr[M(d1) ∈ S] ≤ exp(ε) Pr[M(d2) ∈ S] in ε-DP must be satisfied when specific algorithm M is applied to any data sets d1 and d2 that differ only by 1 row and S means set of all possible outputs of M. ε is called the privacy budget. Privacy protection becomes absolute when ε becomes 0, but the algorithm becomes useless because all predictions will be the same. The performance generally improves as ε increases, but the privacy loss also increases. Thus, we can adjust the trade-off between performance and privacy loss by controlling the amount of ε [26]. DP-SGD applies differential privacy to gradient updates in the training phase rather than applying differential privacy to the trained model. The DP-SGD method clipped the L2 norm of each gradient and added noise with a predefined hyperparameter at each step of the update. DP-SGD has 2 hyperparameters: noise multiplier and multiple gradient norms. We observed the federated model’s mean AUC in 4 development hospitals’ tunning data sets by changing each hyperparameter in the search space of 0.5,1.0, 2.5, and 5.0. We use Rényi differential privacy accountant for privacy loss analysis [27].

### Cross-Site Evaluation

Cross-site evaluation is a process for comparing federated and local models without test data set sharing. We distributed 4 local models and 1 federated model to all clients, calculated the metrics from each client’s test data set, and aggregated the metrics. The cross-site evaluation aimed to show the FL model’s generalizability and noninferior performance versus the local model developed in the test hospital. We compared the federated model versus the mean AUC of the local models in cross-site evaluation. Further, we compared the federated model to the local models developed in the test hospital to show that the federated model is not inferior to the locally specialized model. Furthermore, we externally validated the federated and local models at a geographically and administratively separate hospital. We randomly downsampled patients without BD transition to create a balanced test data set with 1:1, 1:3, 1:4, and 1:9 ratios of patients without outcomes versus patients with outcomes because model performance is sensitive to outcome imbalance. We bootstrapped downsampling 1000 times and calculated the mean AUC and 95% CI in a balanced data set. Additionally, for estimating model calibration, we calculated the Brier score of federated, AUSOM, KHMC, MJ, and KDH models in internal and external test data sets. Brier score is the mean squared difference between predicted probability and the actual outcome; a lower Brier score implies better calibration.

### Statistical Analysis

Data from the 4 development hospitals were divided into 7:1:2 (training, tuning, and test, respectively) on a patient basis. All patient data were used for external validation in the external validation hospital (KW). The summary statistics of the 4 development hospitals were inspected using 2 different methods. First, we used the baseline characteristic table, which is commonly used in clinical studies. Categorical variables were described as percentages and were compared using the *χ*^2^ test for baseline characteristics. *P* values <.05 were considered statistically significant. Second, we used Uniform Manifold Approximation and Projection (UMAP) to visualize the data characteristics of the 4 model development hospitals [28]. UMAP is a visualization technique by dimension reduction. UMAP models manifold data with a fuzzy topological structure and finds a low-dimensional projection embedding with the nearest equivalent fuzzy topological structure. The analyses for cohort creation and feature extraction were performed using R software version 3.6. (The R Project for Statistical Computing), the FeatureExtraction R library of OHDSI, and open-source statistical R packages [29].

The Amazon web service was used for the central server and the local training server in each participating hospital. We used the FeederNet distributed research network. PyTorch 1.8 and Flower 1.1 were used for federated model development. Google Remote Procedure Call was used for network connection. We visualized SHAP (Shapley Additive Explanations) beeswarm plots and bar plots of the federated model and the local model in the research organizing center (AUSOM) for model interpretability [30].

### Ethics Approval

This study was approved by the institutional review board (IRB) of Ajou University Medical Center (AJOUIRB-MDB-2022-255). The IRB waived written informed consent and approved this study. This study uses deidentified CDM data to protect privacy and confidentiality. Therefore, the type and amount of compensation was not part of this study. Following IRB approval at AUSOM, access to KHMC, MJ, KDH, and KW databases was allowed under the IRB mutual recognition agreement (research-free zone agreement). This study complied with the principles of the Declaration of Helsinki.

## Results

### Baseline Characteristics of the Study Population

**Figure 2:**
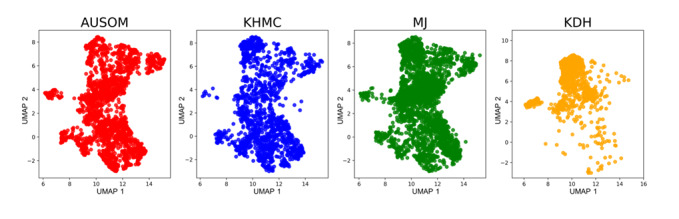
The Uniform Manifold Approximation and Projection of patients with depression according to hospital databases. AUSOM: Ajou University School Of Medicine; KDH: Kangdong Sacred Heart Hospital; KHMC: Kyung Hee Medical Center; MJ: Myongji Hospital; UMAP: Uniform Manifold Approximation and Projection.

For model development, 279 outcomes were used out of 17,631 patient outcomes (AUSOM [outcomes/patients]: 30/3917, KHMC: 58/5799, MJ: 146/4873, and KDH: 45/3042) and for external validation, 39 outcomes were used out of 4541 patient outcomes (KW). Table 1 shows the baseline characteristics of the study participants across all 5 hospitals. The baseline characteristics of each hospital are detailed in Tables S1-S5 in Multimedia Appendix 1. In the age group younger than 40 years, the proportion of patients with bipolar transition was higher than that of patients without bipolar transition. Heart disease and hypertension were more prevalent in patients without bipolar transition. In patients with bipolar transition, severe depression was more prevalent and, in the same vein, mild depression was less prevalent. The distribution of sex between patients with and without bipolar transition revealed no statistical difference. The UMAPs of the development hospitals are detailed in Figure 2. KDH’s UMAP has almost no data projected to the lower middle and upper right region, unlike the UMAP of other hospitals, suggesting the heterogeneity of KDH’s data compared to those of the other hospitals.

**Table 1:** Baseline characteristics of the study population with or without diagnosis transition across all 5 hospitals.

Variable		Nonbipolar (n=21,854), n (%)	Bipolar (n=318), n (%)	Chi-square (*df*)	*P* value
**Age (years)**				9.6 (3)	.02^a^
	<20	998 (4.6)	33 (10.4)		
	20-29	1351 (6.2)	52 (16.4)		
	30-39	2034 (9.3)	38 (11.9)		
	≥40	17,471 (79.9)	195 (61.3)		
Sex (male)		7066 (32.3)	104 (32.7)	0.01 (1)	.94
**Medical history**					
	Diabetes mellitus	1405 (6.4)	15 (4.7)	1.3 (1)	.26
	Heart disease	1399 (6.4)	11 (3.5)	4.1 (1)	.04^a^
	Hypertension	3550 (16.2)	36 (11.3)	5.3 (1)	.02^a^
**Psychiatric history**					
	Anxiety disorder	4499 (20.6)	76 (23.9)	1.9 (1)	.17
	Mild depression	5723 (26.2)	58 (18.2)	9.9 (1)	.002^a^
	Severe depression	1879 (8.6)	54 (17)	26.6 (1)	<.001^a^
	Depression with psychosis	598 (2.7)	3 (0.9)	3.2 (1)	.07
	Substance use disorder	481 (2.2)	5 (1.6)	0.3 (1)	.57
	Suicidal thoughts or self-harm	109 (0.5)	5 (1.6)	5.1 (1)	.02^a^

^a^Significant at *P*<.05.

### Federated Feature Selection

**Figure 3:**
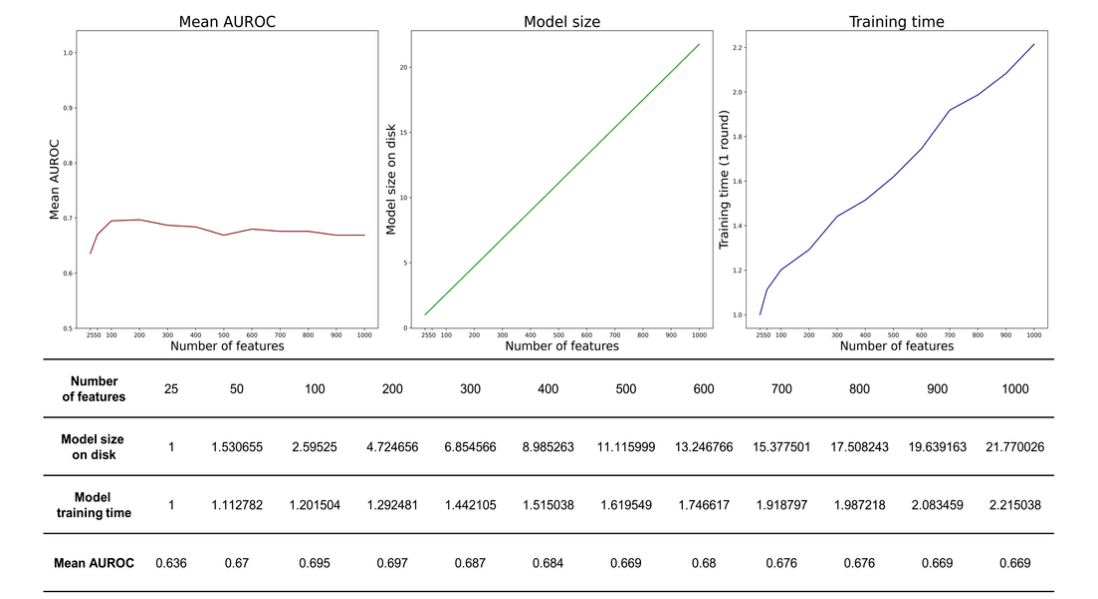
Performance (area under the receiver operating characteristic curve), model size on disk, and training time of the federated model according to the number of features. AUROC: area under the receiver operating characteristic curve.

In our study, the model performance did not increase after n=100. Finally, we developed a model using only 100 out of 21,042 features. Moreover, we calculated the one-epoch training time, the model weight size, and the average performance of the FL model according to N in all search spaces to demonstrate the efficacy of our search method. The model performance could achieve 98% of the highest performance with only n=100. Otherwise, the model weight size and training time increase linearly in proportion to the N. Detailed results are described in Figure 3.

### Privacy-Preserving FL and Cross-Site Evaluation

We set the noise multiplier and multiple gradient norms to 2.5 and 0.5 for DP-SGD because the smallest privacy budget can be achieved with the above parameters while preserving 95% of the model performance compared to the best model. The calculated privacy budget was 1.3 with the above hyperparameter. DP-SGD hyperparameter selection is detailed in Table S6 of Multimedia Appendix 1. Moreover, we developed a local model by using only each client’s training and tuning data sets for comparison with the federated model. The local model was also developed based on only 100 features, the DCN algorithm, and DP-SGD—same as the federated model.

**Figure 4:**
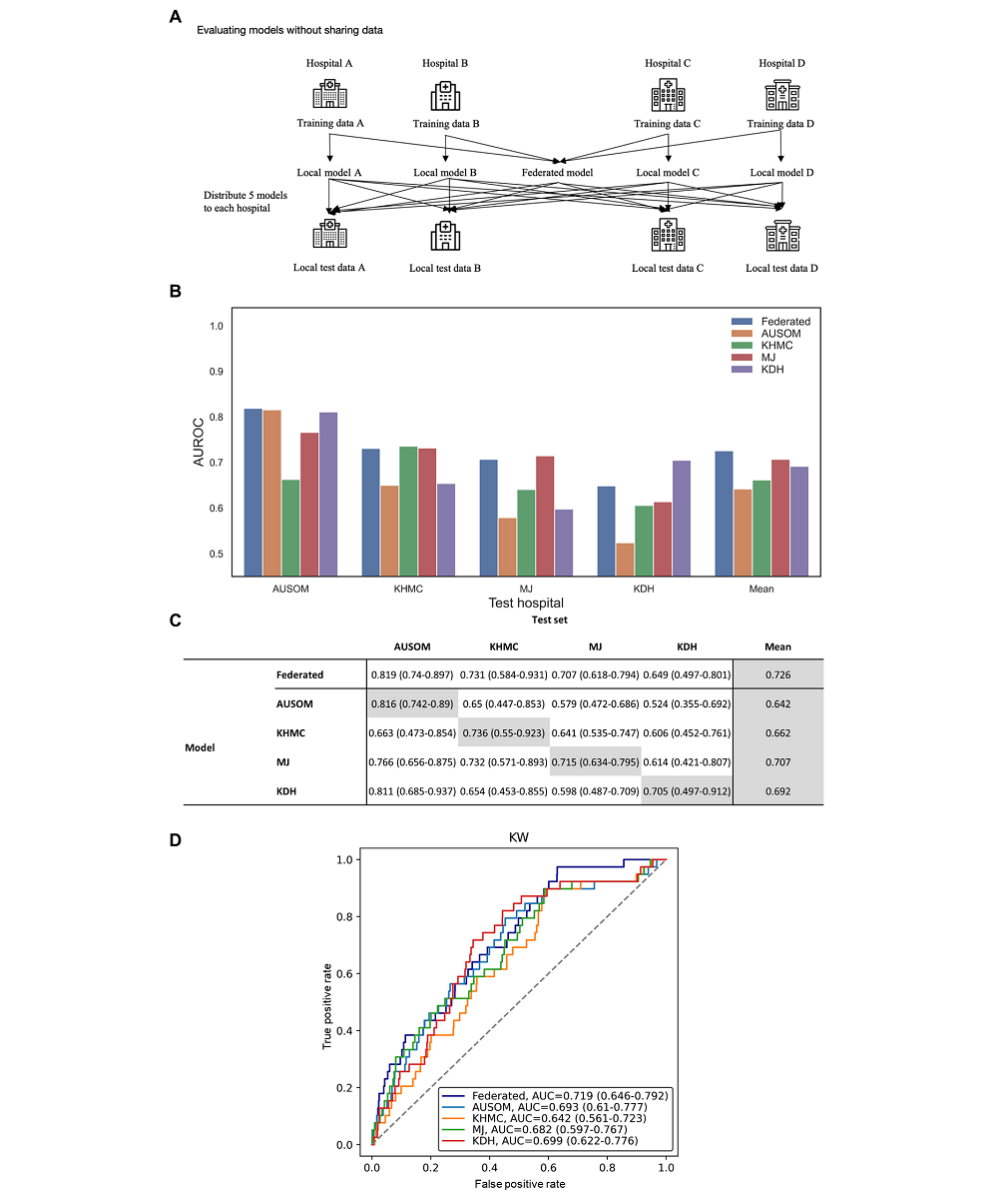
A. Overview of the cross-site evaluation. B. Receiver operating characteristic and mean receiver operating characteristic curves of the federated and local models on development test data sets. C. Performance of external validation using federated learning model and locally trained models. AUC: area under the curve; AUROC: area under the receiver operating characteristic curve; AUSOM: Ajou University School Of Medicine; KDH: Kangdong Sacred Heart Hospital; KHMC: Kyung Hee Medical Center; KW: Kangwon National University Hospital; MJ: Myongji Hospital.

Across all test data sets, the federated model showed a mean AUC of 0.726, while the nonfederated single database models showed mean AUCs of 0.642, 0.662, 0.707, and 0.692 in AUSOM, KHMC, MJ, and KDH, respectively. This suggests the higher generalizability of the federated model than that of any other local model (Figures 4A-4B). Additionally, in the test data set of each client, we compared the performance of the federated model with that of the client’s local model. Calculated AUCs were 0.819 versus 0.816 (federated vs local model, respectively), 0.731 versus 0.736, 0.707 versus 0.715, and 0.649 versus 0.705 in AUSOM, KHMC, MJ, and KDH models, respectively.

The federated model performed better than any other model in external validation (Figure 4C). The AUCs of the federated, AUSOM, KHMC, MJ, and KDH models were 0.719 (95% CI 0.646-0.792), 0.693 (95% CI 0.61-0.777), 0.642 (95% CI 0.561-0.723), 0.682 (95% CI 0.597-0.767), and 0.699 (95% CI 0.622-0.776), respectively.

The average Brier score of the federated model was 0.0150, which was lower than that of AUSOM (0.0218), KHMC (0.0154), MJ (0.0162), and KDH (0.0274). In the external validation, the Brier score of the federated model was 0.009, which was lower than that of AUSOM (0.017), KHMC (0.010), MJ (0.011), and KDH (0.023). Detailed information is described in Figure S1 of Multimedia Appendix 1. As shown in Table S7 of Multimedia Appendix 1, for a balanced test data set, AUC decreased by only 0.007 in AUSOM, 0.001 in MJ, and 0.005 in KDH but increased in KHMC in a federated model evaluation with the balanced data set. Moreover, AUC decreased by only 0.024 in external validation.

### Model Interpretability

**Figure 5:**
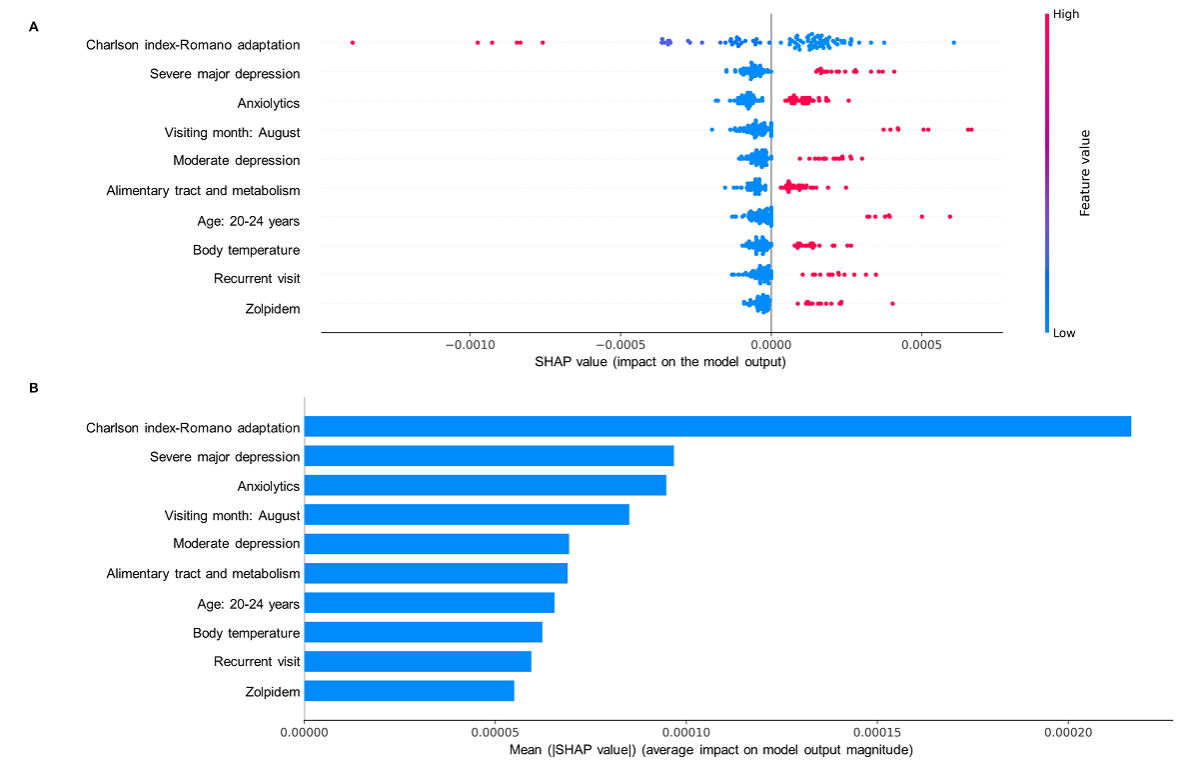
Shapley Additive Explanations beeswarm plot (A) and bar plot (B).

The graphical explanation of the federated model’s top 10 features based on average impact on model output magnitude is shown in Figure 5. The SHAP beeswarm plot is a scatterplot where each point represents the SHAP value of a feature for a particular instance in the data set. The x-axis value of each point represents the SHAP value of that feature for that particular instance. A positive SHAP value indicates an increase in the risk of predicting a diagnostic transition and vice versa. The SHAP bar plot shows the impact of each feature on model prediction. The features are sorted by their importance in the prediction, with the most important feature at the top of the plot. The length of the bar represents the magnitude of the effect on model prediction.

Figure S2 of Multimedia Appendix 1 shows the federated model’s top 20 features. All features used in the federated model are presented in Table S8 of Multimedia Appendix 1. Figures S3-S6 of Multimedia Appendix 1 show the graphical explanations of the local models. In the 2 graphs in Figure 5, the y-axes represent the top 10 features in the prediction model, ranked in the descending order. The x-axis in the SHAP beeswarm plot shows the SHAP value. The SHAP value of each dot means the impact of a feature in the SHAP plot. For example, high median values of severe major depression and moderate depression diagnosis, anxiolytics, alimentary tract and metabolism, zolpidem prescription, visiting month in August, recurrent visit, body temperature, and young age at 20-24 years powerfully drive predictions toward the diagnostic transition from major depressive disorder to BD. Low median values of the Charlson index were more strongly predictive of the diagnostic transition from major depressive disorder to BD (Figure 5A). The types of features were generally similar between the local model and the federated model. However, the impact of the Charlson index feature was higher in the local model (federated model: Figure 5B and Figure S2 of Multimedia Appendix 1; local models: Figures S3-S6 in Multimedia Appendix 1). In addition, antidepressant use was not in the top 10 features in the federated model but was in the top 10 features in the local models, except KDH. In both the federated model and the local models, antidepressant use was associated with a lower risk of bipolar transition (federated model: Figure 5B and Figure S2 of Multimedia Appendix 1; local models: Figures S3-S6 of Multimedia Appendix 1).

## Discussion

### Principal Findings

We developed and validated a bipolar transition prediction model by using distributed multi-institutional psychiatric data in a real-world FL environment. Additionally, we successfully standardized clinical feature extraction pipelines and applied data-driven methods, preserved differential privacy, and extracted clinically interpretable characteristics from the FL model. FL primarily aimed to achieve generalizability. The federated model’s average performance on all test data sets had an AUC of 0.726, which is higher than the 0.642-0.707 of other local models. The FL model performed better than any other model in external validation. Additionally, the FL model was well calibrated compared to any other model in internal and external test data. Consistent performance across multiple validations shows our model’s generalizability. Moreover, our model showed only a minor performance degradation of ≤0.08 compared to the local model trained on that database, except for KDH. The federated model performance dropped by 0.056 compared to the local model performance in KDH, presumably because KDH has a different data distribution from other hospitals, as visualized by UMAP. The model performance degradation in such a nonidentical, independent data set is one of the well-known challenges of FL. However, our model performed better in KDH than any local models developed outside of KDH, suggesting the KDH data with these different distributions contributed sufficiently to the model’s training.

SHAP analysis revealed features with high impacts on models as well-known risk factors. Previous studies reporting the association of diagnostic transition with younger age are consistent with predictive features of the young age group in our federated model [7,31]. A lower Charlson index with BD transition could be explained by the association of BD with age because of the strong positive association between Charlson index and age [32]. The association between severe or moderate depressive episodes with BD transition is in line with other findings that bipolar converters had more severe depressive symptoms than nonconverters [33]. Since there is no depressive symptom itself in the electronic medical record data, the severity was considered as a diagnosis of depression containing symptom information such as moderate depression and severe depression. The results supported anxiolytics among the depression-BD switching predictors in this study that the prevalence of comorbid anxiety disorders in patients with BD was higher than that in patients with depression [34]. Regarding alimentary tract and metabolism, there were significant correlations between patients with BD and gastrointestinal symptoms [35]. Further, somatic symptoms, including gastrointestinal symptoms, tend to co-occur in patients with BD [36]. For the recurrent visit, patients with anxiety appear to be frequent users of medical services [37]. Considering that patients with anxiety have a high rate of bipolar transition, it is likely that they had recurrent visits for the same reason. Sleep disturbance also appears to be an early symptom of BD [38]. Since sleep disturbances often precede BD, zolpidem as a sleep aid could be described as a predictor of bipolar transition. Our study revealed antidepressant use as a predictor of the depression-BD transition risk within 1 year. Additionally, antidepressant use was associated with lower risk, although antidepressant prescriptions were associated with mood cycling and mixed, manic, or hypomanic episodes [39]. The association of antidepressants may be because antidepressant use within 1 year before diagnosis was used as a predictor. People who used antidepressants in advance but did not experience diagnostic transition were included in the study population, while people diagnosed with BD were excluded from this study. Thus, the history of antidepressant use without diagnostic transition could hypothetically have a protective effect on mania/hypomania symptoms. The abovementioned predictors were also reported by Nestsiarovich et al [7]. Likewise, a systematic review of transition from major depression to BD showed that the severity of depression, use of antidepressants, and comorbidity such as anxiety disorder were predictors [40]. However, family history of BD, history of childhood trauma, and history of childhood abuse, which were considered important variables in a systematic review and other studies, were not included in our model [41]. Further, features about temperature and seasonality were not included. A previous study reported that patients with BD had a shorter circadian period and increased body temperature amplitude compared to healthy individuals [42]. For seasonality, patients with BD experienced peak manic symptoms in the spring and summer months, which supports August as a predictor in this study [43]. This study result adds to evidence that variables not present in the existing model could be used as the data-driven model.

One of the major challenges to FL is heterogeneous clinical data. The data extraction process requires individual codes for each hospital, which is resource-consuming and prevents transparent, interoperable, reproducible, and scalable studies. This study used databases converted to OMOP CDM, which is a set of structural and semantic standards about observational data maintained by the OHDSI community [18]. Seventy-four countries have converted or are in the process of converting data to OMOP CDM. The clinical data from 37 hospitals in South Korea were already converted to OMOP CDM [20]. We extracted features with a single analysis code across hospitals by using the same code as the previous study [7] conducted in the United States, with standardized semantics and structure. Through a single code, we extracted large-scale features, thereby creating data-driven prediction models. We published and disclosed our feature extraction settings in GitHub in JSON format following the standard of the OHDSI community for interoperability and transparency. Any medical institution with an OMOP CDM database can reproduce and validate our study. Additionally, it can be extended without code modification if a new hospital wants to participate in training.

Practically, establishing network connections is one of the hardest things in adoption. This study did not build a separate network for only FL. Hospitals in Korea with OMOP CDM databases formed a distributed network called the FeederNet. In this network, patient data were pseudonymized by an honest broker who is a third person other than the researcher [44], and these data were stored in each institution and regularly inspected for data quality. Moreover, each hospital can share aggregated results in real time but not patient-level data. The above network was initially adopted for drug adverse event surveillance. Still, epidemiological analysis is the main interest of health care institutions; thus, building network connections for only FL is challenging. Creating a distributed, federated analytics platform encompassing not only FL but also the epidemiological study will be a practical approach for persuading multi-stakeholders in hospitals.

Hardware insufficiency is another challenge in FL. Our study was conducted only with the CPU in each hospital. We cannot use all the features for training because the number of features extracted in the extraction process was more than 20,000. Accordingly, we reduced the features to 100 through the federated feature selection process and revealed that the model performance could be saturated with only 100 features. Through the process of federated feature selection, we were able to significantly reduce the size of the model while maintaining its performance. As a result, we were able to reduce communication costs, which is one of the biggest challenges in FL, by reducing network latency and overhead caused by weight transferring.

There are 3 primary reasons for decreasing communication costs [45,46]. First, in practice, many institutions lack the necessary computational resources. By reducing the communication overhead, system costs associated with FL can be minimized, thereby lowering the barriers to entry. This allows for a greater diversity of institutions to participate in research and, in the long term, extends the applicability of FL to primary care physicians or patient-generated health data. Second, from a security perspective, limiting the volume of data exchanged during communication improves security measures, as increased data exchange can potentially lead to heightened privacy loss. Homomorphic encryption is regarded as a key security-enhancing algorithm for future FL applications. However, it significantly increases the size of data and computational load. Consequently, the additional overhead caused by such security enhancements can burden even what we consider ample communication resources. Thus, reducing overhead prior to encryption, as proposed in our study, can be beneficial. Third, the benchmarks for sufficient computation and communication resources are continually rising. The advent of developments such as large language models has seen a shift toward foundation models that generate large-scale models based on massive data sets for general purposes. As a result, the required computational power is rapidly increasing. Medical prediction models are also evolving toward these foundation models, thereby dramatically raising the standards for adequate resources. Therefore, additional methods to reduce overhead, like those presented in our study, are necessary. However, our feature selection method included features that were not present that exist in all internal databases. Although this approach was feasible in our study, which was conducted in tertiary hospitals within the same country using unified clinical coding systems, it may be limited in studies involving a wider range of hospitals or countries where the available features vary significantly. Therefore, future studies should explore more comprehensive feature selection methods and consider imputation methods to incorporate features that are missing in some databases.

We conducted a study prepseudonymized by a third-party honest broker to protect patient privacy. The analysis code was written using only aggregated statistics, and the same code was delivered to all institutions for research collaborators to not access patient-level data. We applied differential privacy to limit privacy leakage in the model training to prevent model inversion, which means reconstructing original data from the model gradients [11]. Some shortcomings remained, although we tried to protect privacy through the above steps. We refined the privacy budget when applying differential privacy. However, the appropriate privacy budget has no consensus. We used the strongest privacy while maintaining the model performance of over 95% of maximum performance. However, privacy parameters should be set first, regardless of model performance. Therefore, more differential privacy studies in the medical field should be conducted in the future to find appropriate values.

### Limitations

Our study has a few limitations. First, this study used only tabular data. Therefore, a multimodal model with natural text, genetic data, or image data should be studied for more advanced research in psychiatry. However, natural language may have a more significant privacy leakage hazard. Learning natural language data was practically impossible because only CPUs exist in each hospital. Additionally, genetic data and image data remained not integrated into OMOP CDM databases. Further, we have to conduct a multimodal federated study by developing an efficient architecture.

Second, our study was conducted in only a single nation due to network connection problems. Hence, we conducted external validation at another regionally and administratively separated hospital to compensate for this limitation. However, future studies should include data from other nations because training insufficiency in the nonidentical, independent data set setting is a well-known problem.

Third, our model did not include important but detailed variables such as the family history of BD, history of childhood trauma, and history of childhood abuse. This detailed information may or may not be collected at each hospital. However, because FL uses only common variables for generalization, these important but detailed variables are not used in the prediction model. However, if a hospital has recorded specific information, it can be improved to include specific information by rebuilding the model based on the FL model.

Fourth, the incidence of the outcome of interest in this study was unbalanced (279/17,631, 1.58%). Despite the low incidence, the prediction of bipolar transition has significant clinical value. Although previous researchers such as Nestsiarovich et al [7] and Pradier et al [6] have also developed models using data sets with similar outcome incidences (2.7% and 1.4%, respectively), this study shows the robustness of the model at various incidences through a sensitivity analysis [6,7]. The clinician should be cautious when using this model in clinical settings, as the performance of this model can be greatly influenced by the incidence of the outcome. Future research using various strategies, including refining the cohort, matching clinical variables, and algorithmic adjustments to solve the data set imbalance problem, is needed.

Fifth, we preselected the model algorithm as DCN. Exploring more model architectures would have been better, although DCN showed feasibility for tabular medical data. However, this study could not explore various models because of computer resource limitations. Therefore, we plan to compare the performance of tabular-based models in FL by performing benchmark tests of the performance of several models in the data of this study and an open multi-institutional database.

### Conclusions

In summary, we utilized FL to predict bipolar transition in patients with depression. Bipolar transition could be more effectively predicted using the federated model than a model based only on local data. Furthermore, we used distributed multi-institutional psychiatric data with standardized pipelines in a real-world environment, thereby providing a solution to the challenges of FL, such as data heterogeneity, privacy leakage, and limited computational hospital resources. In clinical situations where data are distributed, our findings suggest that FL would be more useful to develop the prediction model.

## Appendix

Multimedia Appendix 1 Baseline characteristics of each hospital, full results of the federated model, full features of the federated model, and Shapley Additive Explanations plots by federated and local models.

## Abbreviations

AUC: area under the curve
AUSOM: Ajou University School Of Medicine
BD: bipolar disorder
CDM: common data model
CPU: central processing unit
DCN: Deep and Cross Network
DP-SGD: differential private-stochastic gradient descent
FeederNet: Federated E-Health Big Data for Evidence Renovation Network
FL: federated learning
IRB: institutional review board
KDH: Kangdong Sacred Heart Hospital
KHMC: Kyung Hee University Medical Center
KW: Kangwon National University Hospital
MJ: Myongji Hospital
OHDSI: Observational Health Data Sciences and Informatics
OMOP: Observational Medical Outcomes Partnership
SHAP: Shapley Additive Explanations
UMAP: Uniform Manifold Approximation and Projection

## Data Availability

The data sets generated and analyzed during this study are available from the corresponding author upon reasonable request.

## References

[1] GBD 2017 DiseaseInjury Incidence and Prevalence Collaborators (2018). Global, regional, and national incidence, prevalence, and years lived with disability for 354 diseases and injuries for 195 countries and territories, 1990-2017: a systematic analysis for the Global Burden of Disease Study 2017. Lancet.

[2] Angst J (2007). The bipolar spectrum. Br J Psychiatry.

[3] Angst J, Merikangas KR, Cui L, Van Meter A, Ajdacic-Gross V, Rössler W (2018). Bipolar spectrum in major depressive disorders. Eur Arch Psychiatry Clin Neurosci.

[4] Shen H, Zhang Li, Xu Chuchen, Zhu Jinling, Chen Meijuan, Fang Yiru (2018). Analysis of misdiagnosis of bipolar disorder in an outpatient setting. Shanghai Arch Psychiatry.

[5] Merikangas KR, Cui Lihong, Kattan G, Carlson Gabrielle A, Youngstrom Eric A, Angst Jules (2012). Mania with and without depression in a community sample of US adolescents. Arch Gen Psychiatry.

[6] Pradier MF, Hughes MC, McCoy TH, Barroilhet SA, Doshi-Velez F, Perlis RH (2021). Predicting change in diagnosis from major depression to bipolar disorder after antidepressant initiation. Neuropsychopharmacology.

[7] Nestsiarovich A, Reps JM, Matheny ME, DuVall SL, Lynch KE, Beaton M, Jiang X, Spotnitz M, Pfohl SR, Shah NH, Torre CO, Reich CG, Lee DY, Son SJ, You SC, Park RW, Ryan PB, Lambert CG (2021). Predictors of diagnostic transition from major depressive disorder to bipolar disorder: a retrospective observational network study. Transl Psychiatry.

[8] Zerka F, Barakat S, Walsh S, Bogowicz M, Leijenaar RTH, Jochems A, Miraglio B, Townend D, Lambin P (2020). Systematic review of privacy-preserving distributed machine learning from federated databases in health care. JCO Clinical Cancer Informatics.

[9] Mostert M, Koomen B, van Delden J, Bredenoord A (2018). Privacy in big data psychiatric and behavioral research: A multiple-case study. Int J Law Psychiatry.

[10] McMahan B, Moore E, et al Communication-efficient learning of deep networks from decentralized data. arXiv. Preprint posted online on January 26, 2023.

[11] Dayan I, Roth Holger R, Zhong Aoxiao, Harouni Ahmed, Gentili Amilcare, Abidin Anas Z, Liu Andrew, Costa Anthony Beardsworth, Wood Bradford J, Tsai Chien-Sung, Wang Chih-Hung, Hsu Chun-Nan, Lee C K, Ruan Peiying, Xu Daguang, Wu Dufan, Huang Eddie, Kitamura Felipe Campos, Lacey Griffin, de Antônio Corradi Gustavo César, Nino Gustavo, Shin Hao-Hsin, Obinata Hirofumi, Ren Hui, Crane Jason C, Tetreault Jesse, Guan Jiahui, Garrett John W, Kaggie Joshua D, Park Jung Gil, Dreyer Keith, Juluru Krishna, Kersten Kristopher, Rockenbach Marcio Aloisio Bezerra Cavalcanti, Linguraru Marius George, Haider Masoom A, AbdelMaseeh Meena, Rieke Nicola, Damasceno Pablo F, E Silva Pedro Mario Cruz, Wang Pochuan, Xu Sheng, Kawano Shuichi, Sriswasdi Sira, Park Soo Young, Grist Thomas M, Buch Varun, Jantarabenjakul Watsamon, Wang Weichung, Tak Won Young, Li Xiang, Lin Xihong, Kwon Young Joon, Quraini Abood, Feng Andrew, Priest Andrew N, Turkbey Baris, Glicksberg Benjamin, Bizzo Bernardo, Kim Byung Seok, Tor-Díez Carlos, Lee Chia-Cheng, Hsu Chia-Jung, Lin Chin, Lai Chiu-Ling, Hess Christopher P, Compas Colin, Bhatia Deepeksha, Oermann Eric K, Leibovitz Evan, Sasaki Hisashi, Mori Hitoshi, Yang Isaac, Sohn Jae Ho, Murthy Krishna Nand Keshava, Fu Li-Chen, de Mendonça Matheus Ribeiro Furtado, Fralick Mike, Kang Min Kyu, Adil Mohammad, Gangai Natalie, Vateekul Peerapon, Elnajjar Pierre, Hickman Sarah, Majumdar Sharmila, McLeod Shelley L, Reed Sheridan, Gräf Stefan, Harmon Stephanie, Kodama Tatsuya, Puthanakit Thanyawee, Mazzulli Tony, de Lavor Vitor Lima, Rakvongthai Yothin, Lee Yu Rim, Wen Yuhong, Gilbert Fiona J, Flores Mona G, Li Quanzheng (2021). Federated learning for predicting clinical outcomes in patients with COVID-19. Nat Med.

[12] Rieke N, Hancox J, Li W, Milletarì Fausto, Roth HR, Albarqouni S, Bakas S, Galtier MN, Landman BA, Maier-Hein K, Ourselin S, Sheller M, Summers RM, Trask A, Xu D, Baust M, Cardoso MJ (2020). The future of digital health with federated learning. NPJ Digit Med.

[13] Li T, Sahu AK, Talwalkar A, Smith V (2020). Federated learning: challenges, methods, and future directions. IEEE Signal Process Mag.

[14] Ohm P Broken promises of privacy: Responding to the surprising failure of anonymization. University of California Law Review.

[15] Abadi M, Chu A, et al (2016). Deep learning with differential privacy.

[16] Remeseiro B, Bolon-Canedo V (2019). A review of feature selection methods in medical applications. Comput Biol Med.

[17] Hripcsak George, Duke Jon D, Shah Nigam H, Reich Christian G, Huser Vojtech, Schuemie Martijn J, Suchard Marc A, Park Rae Woong, Wong Ian Chi Kei, Rijnbeek Peter R, van der Lei Johan, Pratt Nicole, Norén G Niklas, Li Yu-Chuan, Stang Paul E, Madigan David, Ryan Patrick B (2015). Observational Health Data Sciences and Informatics (OHDSI): opportunities for observational researchers. Stud Health Technol Inform.

[18] Zhang X, Wang L, Miao S, Xu H, Yin Y, Zhu Y, Dai Z, Shan T, Jing S, Wang J, Zhang X, Huang Z, Wang Z, Guo J, Liu Y (2018). Analysis of treatment pathways for three chronic diseases using OMOP CDM. J Med Syst.

[19] Observational Health Data Sciences and Informatics.

[20] Byun JungHyun, Lee Dong Yun, Jeong Chang-Won, Kim Yerim, Rhee Hak Young, Moon Ki Won, Heo Jeongwon, Hong Yoonki, Kim Woo Jin, Nam Seung-Joo, Choi Hoon Sung, Park Ji In, Chun In Kook, Bak So Hyeon, Lee Kyoungyul, Byeon Gi Hwan, Kim Kyoung Lae, Kim Jeong-Ah, Park Young Joo, Kim Jeong Hyun, Lee Eun Ju, Lee Sang-Ah, Kwon Sung Ok, Park Sang-Won, Kasani Payam Hosseinzadeh, Kim Jung-Kyeom, Kim Yeshin, Kim Seongheon, Jang Jae-Won (2022). Analysis of treatment pattern of anti-dementia medications in newly diagnosed Alzheimer's dementia using OMOP CDM. Sci Rep.

[21] Huser V, Li Xiaochun, Zhang Zuoyi, Jung Sungjae, Park Rae Woong, Banda Juan, Razzaghi Hanieh, Londhe Ajit, Natarajan Karthik (2019). Extending Achilles heel data quality tool with new rules informed by multi-site data quality comparison. Stud Health Technol Inform.

[22] Dixon BE, Wen C, French T, Williams JL, Duke JD, Grannis SJ (2020). Extending an open-source tool to measure data quality: case report on Observational Health Data Science and Informatics (OHDSI). BMJ Health Care Inform.

[23] Federated patient level prediction. GitHub.

[24] Ke  G, Meng Q, Finley T, Wang T, Chen W, Ma Weidong, Ye Q, Liu T-Y (2017). LightGBM: A Highly Efficient Gradient Boosting Decision Tree. Advances in Neural Information Processing Systems 30 (NIPS 2017).

[25] Wang R, Fu B, et al (2017).

[26] Dwork C, Roth A (2014). The algorithmic foundations of differential privacy. FNT in Theoretical Computer Science.

[27] Wang Y, Balle B, Kasiviswanathan S Subsampled Rényi differential privacy and analytical moments accountant. arXiv. Preprint posted online on December 4, 2018.

[28] McInnes L, Healy J, Saul N, Großberger L (2018). UMAP: Uniform Manifold Approximation and Projection. JOSS.

[29] Reps J, Schuemie Martijn J, Suchard Marc A, Ryan Patrick B, Rijnbeek Peter R (2018). Design and implementation of a standardized framework to generate and evaluate patient-level prediction models using observational healthcare data. J Am Med Inform Assoc.

[30] Lundberg S, Lee S A unified approach to interpreting model predictions. arXiv. Preprint posted online on November 25, 2017.

[31] Othmer E, Desouza Cherilyn M, Penick EC, Nickel EJ, Hunter EE, Othmer SC, Powell BJ, Hall SB (2007). Indicators of mania in depressed outpatients: a retrospective analysis of data from the Kansas 1500 study. J Clin Psychiatry.

[32] Khan NF, Perera R, Harper S, Rose PW (2010). Adaptation and validation of the Charlson Index for Read/OXMIS coded databases. BMC Fam Pract.

[33] Goldberg JF, Harrow M, Whiteside JE (2001). Risk for bipolar illness in patients initially hospitalized for unipolar depression. Am J Psychiatry.

[34] Inoue T, Kimura T, Inagaki Y, Shirakawa O (2020). Prevalence of comorbid anxiety disorders and their associated factors in patients with bipolar disorder or major depressive disorder. NDT.

[35] Karling P, Maripuu M, Wikgren M, Adolfsson R, Norrback K (2016). Association between gastrointestinal symptoms and affectivity in patients with bipolar disorder. World J Gastroenterol.

[36] Edgcomb JB, Tseng C, Kerner B (2016). Medically unexplained somatic symptoms and bipolar spectrum disorders: a systematic review and meta-analysis. J Affect Disord.

[37] Deacon B, Lickel J, Abramowitz JS (2008). Medical utilization across the anxiety disorders. J Anxiety Disord.

[38] Ritter P, Marx Carolin, Bauer Michael, Leopold Karolina, Pfennig Andrea (2011). The role of disturbed sleep in the early recognition of bipolar disorder: a systematic review. Bipolar Disord.

[39] Pacchiarotti I, Bond David J, Baldessarini Ross J, Nolen Willem A, Grunze Heinz, Licht Rasmus W, Post Robert M, Berk Michael, Goodwin Guy M, Sachs Gary S, Tondo Leonardo, Findling Robert L, Youngstrom Eric A, Tohen Mauricio, Undurraga Juan, González-Pinto Ana, Goldberg Joseph F, Yildiz Ayşegül, Altshuler Lori L, Calabrese Joseph R, Mitchell Philip B, Thase Michael E, Koukopoulos Athanasios, Colom Francesc, Frye Mark A, Malhi Gin S, Fountoulakis Konstantinos N, Vázquez Gustavo, Perlis Roy H, Ketter Terence A, Cassidy Frederick, Akiskal Hagop, Azorin Jean-Michel, Valentí Marc, Mazzei Diego Hidalgo, Lafer Beny, Kato Tadafumi, Mazzarini Lorenzo, Martínez-Aran Anabel, Parker Gordon, Souery Daniel, Ozerdem Ayşegül, McElroy Susan L, Girardi Paolo, Bauer Michael, Yatham Lakshmi N, Zarate Carlos A, Nierenberg Andrew A, Birmaher Boris, Kanba Shigenobu, El-Mallakh Rif S, Serretti Alessandro, Rihmer Zoltan, Young Allan H, Kotzalidis Georgios D, MacQueen Glenda M, Bowden Charles L, Ghaemi S Nassir, Lopez-Jaramillo Carlos, Rybakowski Janusz, Ha Kyooseob, Perugi Giulio, Kasper Siegfried, Amsterdam Jay D, Hirschfeld Robert M, Kapczinski Flávio, Vieta Eduard (2013). The International Society for Bipolar Disorders (ISBD) task force report on antidepressant use in bipolar disorders. Am J Psychiatry.

[40] Ratheesh A, Davey C, Hetrick S, Alvarez-Jimenez M, Voutier C, Bechdolf A, McGorry PD, Scott J, Berk M, Cotton SM (2017). A systematic review and meta-analysis of prospective transition from major depression to bipolar disorder. Acta Psychiatr Scand.

[41] Musliner KL, Østergaard SD (2018). Patterns and predictors of conversion to bipolar disorder in 91 587 individuals diagnosed with unipolar depression. Acta Psychiatr Scand.

[42] Dallaspezia S, Benedetti F (2015). Chronobiology of bipolar disorder: therapeutic implication. Curr Psychiatry Rep.

[43] Geoffroy PA, Bellivier F, Scott J, Etain B (2014). Seasonality and bipolar disorder: a systematic review, from admission rates to seasonality of symptoms. J Affect Disord.

[44] Boyd AD, Hosner C, Hunscher DA, Athey BD, Clauw DJ, Green LA (2007). An 'Honest Broker' mechanism to maintain privacy for patient care and academic medical research. Int J Med Inform.

[45] Sheller M, Reina G Anthony, Edwards Brandon, Martin Jason, Bakas Spyridon (2019). Multi-institutional deep learning modeling without sharing patient data: a feasibility study on brain tumor segmentation. Brainlesion.

[46] Brisimi TS, Chen R, Mela T, Olshevsky A, Paschalidis IC, Shi W (2018). Federated learning of predictive models from federated Electronic Health Records. Int J Med Inform.

