# Supplementary material for: Privacy-Preserving Federated Model Predicting Bipolar Transition in Patients With Depression: Prediction Model Development Study
Source: J Med Internet Res. 2023 Jul 20;25:e46165. doi: 10.2196/46165 (PMC10401196; doi:10.2196/46165)

**Table S1**. Baseline characteristics for study population with or without diagnosis transition in AUSOM

| **Variable** | **Non-bipolar**  **(n = 3,887)** | **Bipolar**  **(n = 30)** | $\boldsymbol{\chi}_{\left( \boldsymbol{df} \right)}^{\boldsymbol{2}}$ | **P-value** | |
| --- | --- | --- | --- | --- | --- |
| **Age group, n (%)** |  |  | 7.86(3) | 0.04***** | |
| < 20 | 253 (6.5) | 4 (13.3) |  |  | |
| 20 – 29 | 334 (8.6) | 5 (16.7) |  |  | |
| 30 – 39 | 595 (15.3) | 7 (23.3) |  |  | |
| ≥ 40 | 2,705 (69.6) | 14 (46.7) |  |  | |
| **Sex, n (%)** | | | | |  |
| Male | 1,166 (30.0) | 7 (23.3) | 0.35(1) | 0.55 | |
| **Medical history, n (%)** | | | | |  |
| Diabetes mellitus | 222 (5.7) | 1 (3.3) | 0.03(1) | 0.87 | |
| Heart disease | 241 (6.2) | 2 (6.7) | <0.01(1) | 1.00 | |
| Hypertension | 346 (8.9) | 0 (0.0) | 1.93(1) | 0.17 | |
| **Psychiatric history, n (%)** | | | | |  |
| Anxiety disorder | 466 (12.0) | 3 (10.0) | <0.01(1) | 0.95 | |
| Mild depression | 334 (8.6) | 1 (3.3) | 0.49(1) | 0.48 | |
| Severe depression | 882 (22.7) | 14 (46.7) | 8.39(1) | <0.01* | |
| depression with psychosis | 533 (13.7) | 3 (10.0) | 0.10(1) | 0.75 | |
| Substance use disorder | 74 (1.9) | 1 (3.3) | <0.01(1) | 1.00 | |
| Suicidal thoughts or self-harm | 77 (2.0) | 2 (6.6) | 1.36(1) | 0.24 | |

$\chi_{\left( df \right)}^{2}$: *chi-square value and degree of freedom; * indicates statistical significance (P-value < 0.05)*

**Table S2**. Baseline characteristics for study population with or without diagnosis transition in KHMC

| **Variable** | **Non-bipolar**  **(n = 5,741)** | **Bipolar**  **(n = 58)** | $\boldsymbol{\chi}_{\left( \boldsymbol{df} \right)}^{\boldsymbol{2}}$ | **P-value** | |
| --- | --- | --- | --- | --- | --- |
| **Age group, n (%)** |  |  | 24.96(3) | <0.01***** | |
| < 20 | 97 (1.7) | 6 (10.3) |  |  | |
| 20 – 29 | 283 (4.9) | 10 (17.2) |  |  | |
| 30 – 39 | 296 (5.2) | 9 (15.6) |  |  | |
| ≥ 40 | 5,065 (88.2) | 33 (56.9) |  |  | |
| **Sex, n (%)** | | | | |  |
| Male | 1,804 (31.4) | 19 (32.8) | 0.01(1) | 0.94 | |
| **Medical history, n (%)** | | | | |  |
| Diabetes mellitus | 700 (12.2) | 4 (6.9) | 1.05(1) | 0.30 | |
| Heart disease | 590 (10.3) | 2 (3.4) | 2.22(1) | 0.13 | |
| Hypertension | 1,635 (28.5) | 7 (12.1) | 6.83(1) | <0.01* | |
| **Psychiatric history, n (%)** | | | | |  |
| Anxiety disorder | 2,051 (35.7) | 15 (25.9) | 2.02(1) | 0.16 | |
| Mild depression | 1,554 (27.1) | 9 (15.5) | 3.33(1) | 0.07 | |
| Severe depression | 148 (2.6) | 4 (6.9) | 2.67(1) | 0.10 | |
| depression with psychosis | 9 (0.2) | 0 (0.0) | <0.01(1) | 1.00 | |
| Substance use disorder | 83 (1.4) | 2 (3.4) | 0.51(1) | 0.48 | |
| Suicidal thoughts or self-harm | 9 (0.2) | 1 (1.7) | 1.62(1) | 0.20 | |

$\chi_{\left( df \right)}^{2}$: *chi-square value and degree of freedom; * indicates statistical significance (P-value < 0.05)*

**Table S3**. Baseline characteristics for study population with or without diagnosis transition in MJ

| **Variable** | **Non-bipolar**  **(n = 4,727)** | **Bipolar**  **(n = 146)** | $\boldsymbol{\chi}_{\left( \boldsymbol{df} \right)}^{\boldsymbol{2}}$ | **P-value** | |
| --- | --- | --- | --- | --- | --- |
| **Age group, n (%)** |  |  | 2.99(3) | 0.39 | |
| < 20 | 251 (5.3) | 5 (3.4) |  |  | |
| 20 – 29 | 270 (5.7) | 18 (12.3) |  |  | |
| 30 – 39 | 446 (9.4) | 14 (9.6) |  |  | |
| ≥ 40 | 3,760 (79.6) | 109 (74.7) |  |  | |
| **Sex, n (%)** | | | | |  |
| Male | 1,620 (34.3) | 50 (34.2) | <0.01(1) | 1.00 | |
| **Medical history, n (%)** | | | | |  |
| Diabetes mellitus | 314 (6.6) | 10 (6.8) | <0.01(1) | 1.00 | |
| Heart disease | 297 (6.3) | 6 (4.1) | 0.80(1) | 0.37 | |
| Hypertension | 842 (17.8) | 27 (18.5) | 0.01(1) | 0.91 | |
| **Psychiatric history, n (%)** | | | | |  |
| Anxiety disorder | 1,029 (21.8) | 49 (33.6) | 10.76(1) | <0.01* | |
| Mild depression | 2120 (44.8) | 44 (30.1) | 11.83(1) | <0.01* | |
| Severe depression | 219 (4.6) | 21 (14.4) | 26.71(1) | <0.01* | |
| depression with psychosis | 15 (0.3) | 0 (0.0) | <0.01(1) | 1.00 | |
| Substance use disorder | 92 (1.9) | 1 (0.7) | 0.62(1) | 0.43 | |
| Suicidal thoughts or self-harm | 8 (0.2) | 0 (0.0) | <0.01(1) | 1.00 | |

$\chi_{\left( df \right)}^{2}$: *chi-square value and degree of freedom; * indicates statistical significance (P-value < 0.05)*

**Table S4**. Baseline characteristics for study population with or without diagnosis transition in KDH

| **Variable** | **Non-bipolar**  **(n = 2,997)** | **Bipolar**  **(n = 45)** | $\boldsymbol{\chi}_{\left( \boldsymbol{df} \right)}^{\boldsymbol{2}}$ | **P-value** | |
| --- | --- | --- | --- | --- | --- |
| **Age group, n (%)** |  |  | 13.25(3) | <0.01***** | |
| < 20 | 106 (3.5) | 3 (6.7) |  |  | |
| 20 – 29 | 188 (6.3) | 10 (22.2) |  |  | |
| 30 – 39 | 341 (11.4) | 6 (13.3) |  |  | |
| ≥ 40 | 2,362 (78.8) | 26 (57.8) |  |  | |
| **Sex, n (%)** | | | | |  |
| Male | 869 (29.0) | 13 (28.9) | <0.01(1) | 1.00 | |
| **Medical history, n (%)** | | | | |  |
| Diabetes mellitus | 113 (3.8) | 0 (0.0) | 0.87(1) | 0.35 | |
| Heart disease | 99 (3.3) | 0 (0.0) | 0.67(1) | 0.41 | |
| Hypertension | 250 (8.3) | 2 (4.4) | 0.45(1) | 0.50 | |
| **Psychiatric history, n (%)** | | | | |  |
| Anxiety disorder | 394 (13.1) | 5 (11.1) | 0.03(1) | 0.86 | |
| Mild depression | 175 (5.8) | 0 (0.0) | 1.82(1) | 0.18 | |
| Severe depression | 459 (15.3) | 8 (17.8) | 0.06(1) | 0.81 | |
| depression with psychosis | 25 (0.8) | 0 (0.0) | <0.01(1) | 1.00 | |
| Substance use disorder | 84 (2.8) | 1 (2.2) | <0.01(1) | 1.00 | |
| Suicidal thoughts or self-harm | 2 (0.1) | 0 (0.0) | <0.01(1) | 1.0 | |

$\chi_{\left( df \right)}^{2}$: *chi-square value and degree of freedom; * indicates statistical significance (P-value < 0.05)*

**Table S5**. Baseline characteristics for study population with or without diagnosis transition in KW

| **Variable** | **Non-bipolar**  **(n = 4,502)** | **Bipolar**  **(n = 39)** | $\boldsymbol{\chi}_{\left( \boldsymbol{df} \right)}^{\boldsymbol{2}}$ | **P-value** | |
| --- | --- | --- | --- | --- | --- |
| **Age group, n (%)** |  |  | 52.18(3) | <0.01***** | |
| < 20 | 291 (6.5) | 15 (38.5) |  |  | |
| 20 – 29 | 276 (6.1) | 9 (23.1) |  |  | |
| 30 – 39 | 356 (7.9) | 2 (5.1) |  |  | |
| ≥ 40 | 3,579 (79.5) | 13 (33.3) |  |  | |
| **Sex, n (%)** | | | | |  |
| Male | 1,607 (35.7) | 15 (38.5) | 0.04(1) | 0.85 | |
| **Medical history, n (%)** | | | | |  |
| Diabetes mellitus | 56 (1.2) | 0 (0.0) | <0.01(1) | 1.00 | |
| Heart disease | 172 (3.8) | 1 (2.6) | <0.01(1) | 1.00 | |
| Hypertension | 477 (10.6) | 0 (0.0) | 3.56(1) | 0.06 | |
| **Psychiatric history, n (%)** | | | | |  |
| Anxiety disorder | 559 (12.4) | 4 (10.3) | 0.03(1) | 0.87 | |
| Mild depression | 1,540 (34.2) | 4 (10.3) | 8.85(1) | <0.01* | |
| Severe depression | 171 (3.8) | 7 (17.9) | 16.97(1) | <0.01* | |
| depression with psychosis | 16 (0.4) | 0 (0.0) | <0.01(1) | 1.00 | |
| Substance use disorder | 148 (3.3) | 0 (0.0) | 0.49 | 0.48 | |
| Suicidal thoughts or self-harm | 13 (0.3) | 2 (5.1) | 14.77(1) | <0.01* | |

$\chi_{\left( df \right)}^{2}$: *chi-square value and degree of freedom; * indicates statistical significance (P-value < 0.05)*

**Table S6**. Performances with different differential privacy parameters

| Noise multiplier | 0.5 | | | | 1 | | | | 2.5 | | | | 5 | | | |
| --- | --- | --- | --- | --- | --- | --- | --- | --- | --- | --- | --- | --- | --- | --- | --- | --- |
| Multiple gradient norm | 0.5 | 1 | 2.5 | 5 | 0.5 | 1 | 2.5 | 5 | 0.5 | 1 | 2.5 | 5 | 0.5 | 1 | 2.5 | 5 |
| Maximum privacy budget | 14.39 | | | | 4.74 | | | | 1.3 | | | | 0.56 | | | |
| Mean AUROC | 0.707 | 0.732 | 0.732 | 0.733 | 0.698 | 0.732 | 0.732 | 0.733 | 0.501 | 0.551 | 0.707 | 0.726 | 0.484 | 0.482 | 0.595 | 0.707 |

*Note.* Noise multiplier and multiple gradient norm is a hyperparameter of differential private stochastic gradient descent.

We show the changes in Noise Multiplier and Multiple Gradient Norm, two hyperparameters of DP-SGD, and the corresponding maximum privacy budget. Additionally, we have included the metric of the model under the maximum privacy budget. From this table, we can observe a decrease in performance with a smaller privacy budget. This is consistent with the common knowledge that stronger privacy restrictions can lead to a decrease in model performance.

**Table S7. Federated model performance with balanced test dataset**

|  | The ratio between patients with outcome versus without outcome | | | |
| --- | --- | --- | --- | --- |
|  | 1:1 | 1:3 | 1:4 | 1:9 |
| AUSOM | 0.809(0.561-1.058) | 0.817(0.667-0.967) | 0.813(0.695-0.930) | 0.815(0.734-0.896) |
| KHMC | 0.761(0.647-0.876) | 0.755(0.689-0.821) | 0.757(0.695-0.819) | 0.759(0.725-0.794) |
| MJ | 0.707(0.610-0.804) | 0.706(0.654-0.757) | 0.710(0.662-0.758) | 0.709(0.681-0.737) |
| KDH | 0.644(0.445-0.843) | 0.654(0.551-0.758) | 0.661(0.565-0.757) | 0.662(0.602-0.721) |
| KW | 0.695(0.607-0.784) | 0.698(0.648-0.747) | 0.695(0.655-0.736) | 0.701(0.677-0.725) |

We conducted this sensitivity analysis to verify the real applicability of the model. The actual environment in which the model is applied may differ from the training and validation environments we used. Especially, differences in outcome incidence can significantly affect the model's performance. Therefore, we believed it was essential to demonstrate that the model's performance is robust even if there are changes in the outcome incidence in the dataset.

**Table S8. All features used in federated model**

| Features |
| --- |
| age : 10 - 14 |
| age : 15 - 19 |
| age : 20 - 24 |
| age : 25 - 29 |
| age : 30 - 34 |
| age : 35 - 39 |
| age : 40 - 44 |
| age : 45 - 49 |
| age : 50 - 54 |
| age : 55 - 59 |
| age : 60 - 64 |
| age : 65 - 69 |
| age : 70 - 74 |
| age : 75 - 79 |
| charlson index - romano adaptation |
| condition_era , last 30 days: acute stress disorder |
| condition_era , last 30 days: adjustment disorder |
| condition_era , last 30 days: anxiety |
| condition_era , last 30 days: disorders of initiating and maintaining sleep |
| condition_era , last 30 days: dyssomnia |
| condition_era , last 30 days: dysthymia |
| condition_era , last 30 days: finding related to sleep |
| condition_era , last 30 days: inflammation of specific body organs |
| condition_era , last 30 days: inflammation of specific body systems |
| condition_era , last 30 days: mild depression |
| condition_era , last 30 days: mixed anxiety and depressive disorder |
| condition_era , last 30 days: moderate depression |
| condition_era , last 30 days: non-organic sleep disorder |
| condition_era , last 30 days: pain |
| condition_era , last 30 days: recurrent disease |
| condition_era , last 30 days: recurrent major depression |
| condition_era , last 30 days: severe major depression |
| condition_era , last 365 days: adjustment disorder |
| condition_era , last 365 days: anxiety |
| condition_era , last 365 days: ear, nose and throat disorder |
| condition_era , last 365 days: esophagitis |
| condition_era , last 365 days: finding related to sleep |
| condition_era , last 365 days: hypertensive disorder |
| condition_era , last 365 days: hypothyroidism |
| condition_era , last 365 days: inflammation of specific body systems |
| condition_era , last 365 days: pain |
| drug_era , last 30 days: alimentary tract and metabolism |
| drug_era , last 30 days: anxiolytics |
| drug_era , last 30 days: cardiovascular system disorder |
| drug_era , last 30 days: other antidepressants |
| drug_era , last 30 days: zolpidem |
| drug_era , last 365 days: alimentary tract and metabolism |
| drug_era , last 365 days: alprazolam |
| drug_era , last 365 days: antiepileptics |
| drug_era , last 365 days: antiinflammatory and antirheumatic products, non-steroids |
| drug_era , last 365 days: antipsychotics |
| drug_era , last 365 days: anxiolytics |
| drug_era , last 365 days: drugs for acid related disorders |
| drug_era , last 365 days: drugs for peptic ulcer and gastro-oesophageal reflux disease (gord) |
| drug_era , last 365 days: escitalopram |
| drug_era , last 365 days: hypnotics and sedatives |
| drug_era , last 365 days: lorazepam |
| drug_era , last 365 days: mirtazapine |
| drug_era , last 365 days: musculo-skeletal system |
| drug_era , last 365 days: nervous system disorder |
| drug_era , last 365 days: other analgesics and antipyretics |
| drug_era , last 365 days: other antidepressants |
| drug_era , last 365 days: paroxetine |
| drug_era , last 365 days: selective serotonin reuptake inhibitors |
| drug_era , last 365 days: sensory organs disorder |
| drug_era , last 365 days: sertraline |
| drug_era , last 365 days: trazodone |
| drug_era on the visit day: alimentary tract and metabolism |
| drug_era on the visit day: anxiolytics |
| drug_era on the visit day: drugs for acid related disorders |
| drug_era on the visit day: hypnotics and sedatives |
| drug_era on the visit day: lorazepam |
| drug_era on the visit day: mosapride |
| drug_era on the visit day: nervous system disorder |
| drug_era on the visit day: zolpidem |
| gender = female |
| gender = male |
| measurement above normal range, last 365 days: urate [mass/volume] in serum or plasma |
| measurement below normal range, last 365 days: urate [mass/volume] in serum or plasma |
| measurement below normal range, last 365 days: urea nitrogen serum/plasma |
| measurement below normal range, last 365 days: hemoglobin [mass/volume] in serum or plasma |
| measurement below normal range, last 365 days: hemoglobin serum/plasma |
| measurement, last 30 days: erythrocyte sedimentation rate |
| measurement, last 365 days: body temperature |
| measurement, last 365 days: cholesterol in ldl [mass/volume] in serum or plasma |
| observation, last 30 days: recurrent visit |
| observation, last 365 days: recurrent visit |
| procedure_occurrence, last 365 days: plain chest x-ray |
| visiting month: 1 |
| visiting month: 10 |
| visiting month: 11 |
| visiting month: 12 |
| visiting month: 2 |
| visiting month: 3 |
| visiting month: 4 |
| visiting month: 5 |
| visiting month: 6 |
| visiting month: 7 |
| visiting month: 8 |
| visiting month: 9 |

**Figure S1. Calculated Brier score for comparing the calibration of FL- and locally trained models**

**
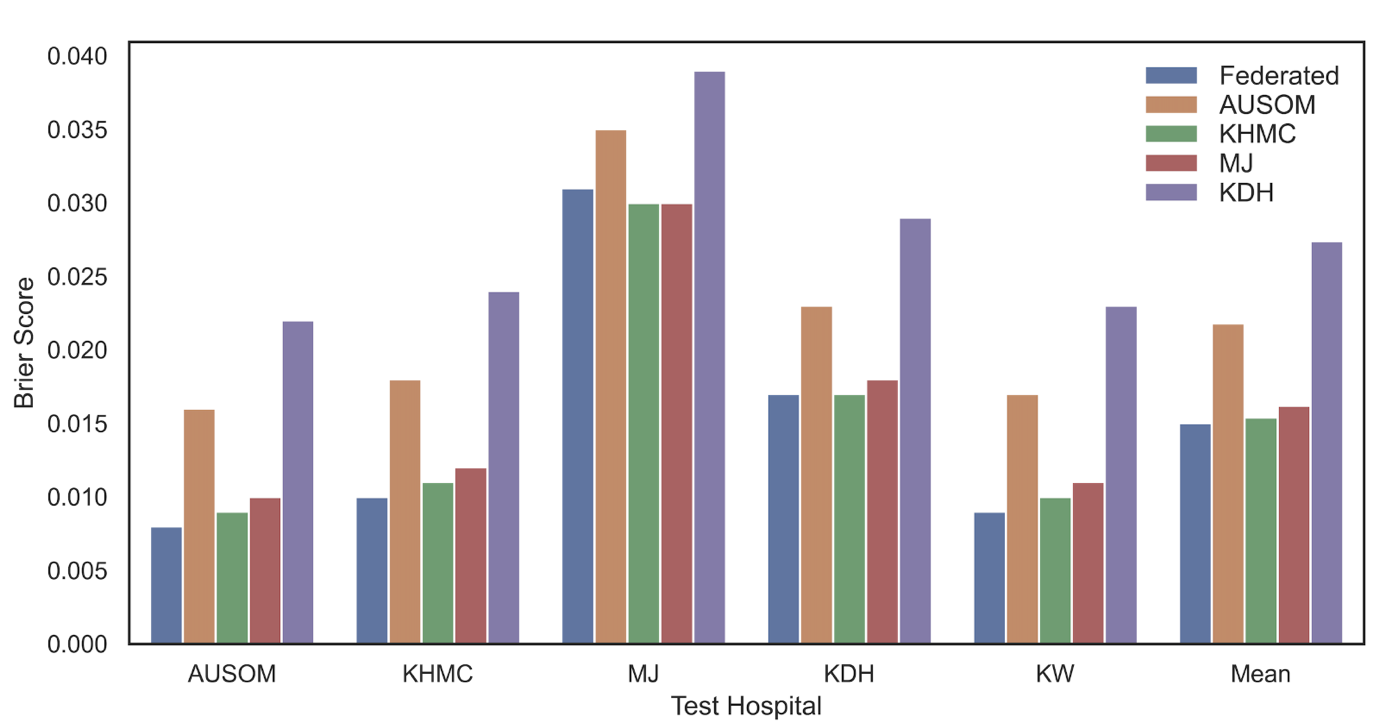
**

|  |  | Data  (internal) | | | | Data (external) |  |
| --- | --- | --- | --- | --- | --- | --- | --- |
|  |  | AUSOM | KHMC | MJ | KDH | KW | Mean |
| Model | Federated | 0.008 | 0.010 | 0.031 | 0.017 | 0.009 | 0.0150 |
|  | AUSOM | 0.016 | 0.018 | 0.035 | 0.023 | 0.017 | 0.0218 |
|  | KHMC | 0.009 | 0.011 | 0.030 | 0.017 | 0.010 | 0.0154 |
|  | MJ | 0.010 | 0.012 | 0.030 | 0.018 | 0.011 | 0.0162 |
|  | KDH | 0.022 | 0.024 | 0.039 | 0.029 | 0.023 | 0.0274 |

**Figure S2. SHAP plots of the federated model with top 20 features**


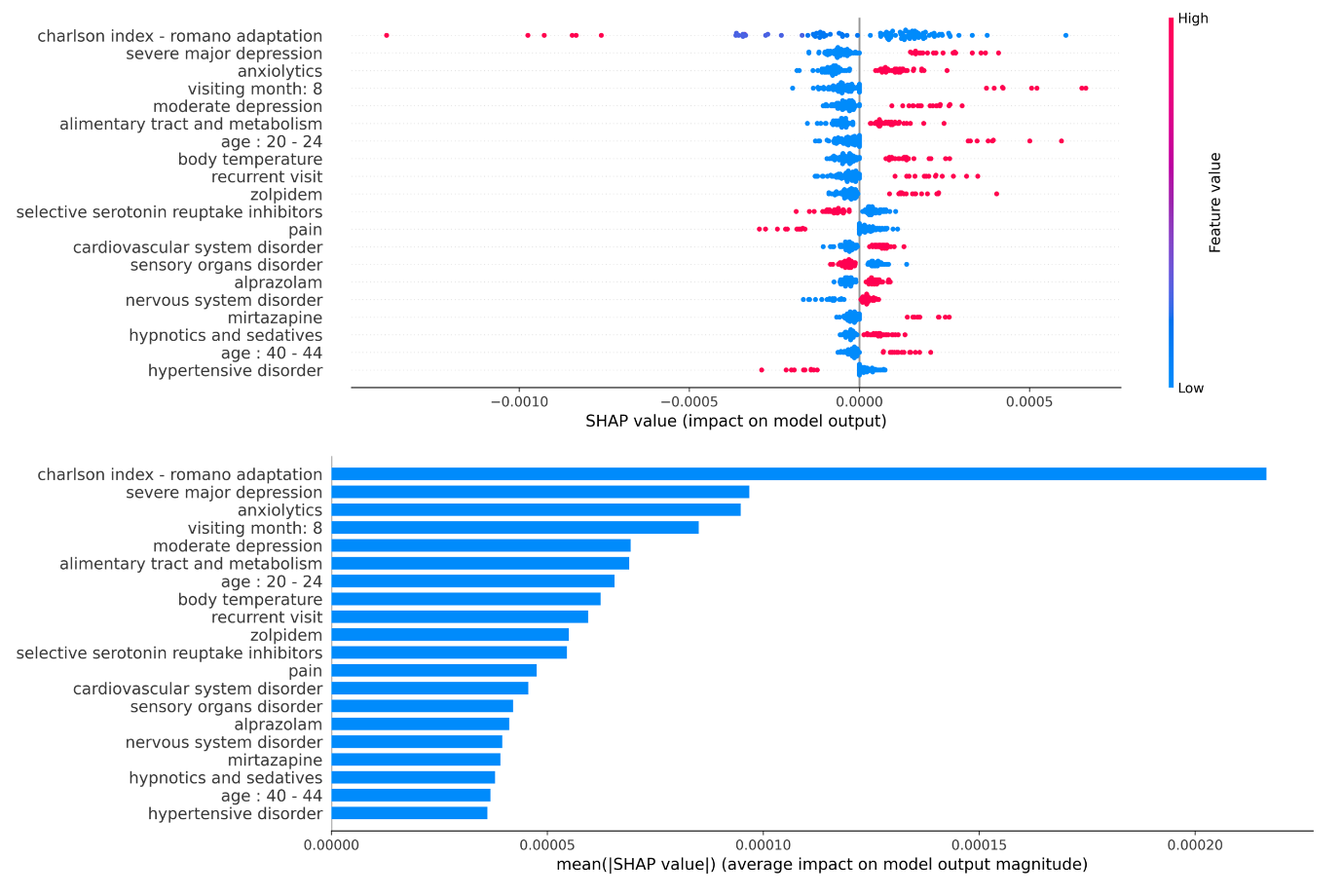


**Figure S3. SHAP plots of the local model in the Ajou University School Of Medicine (AUSOM)**


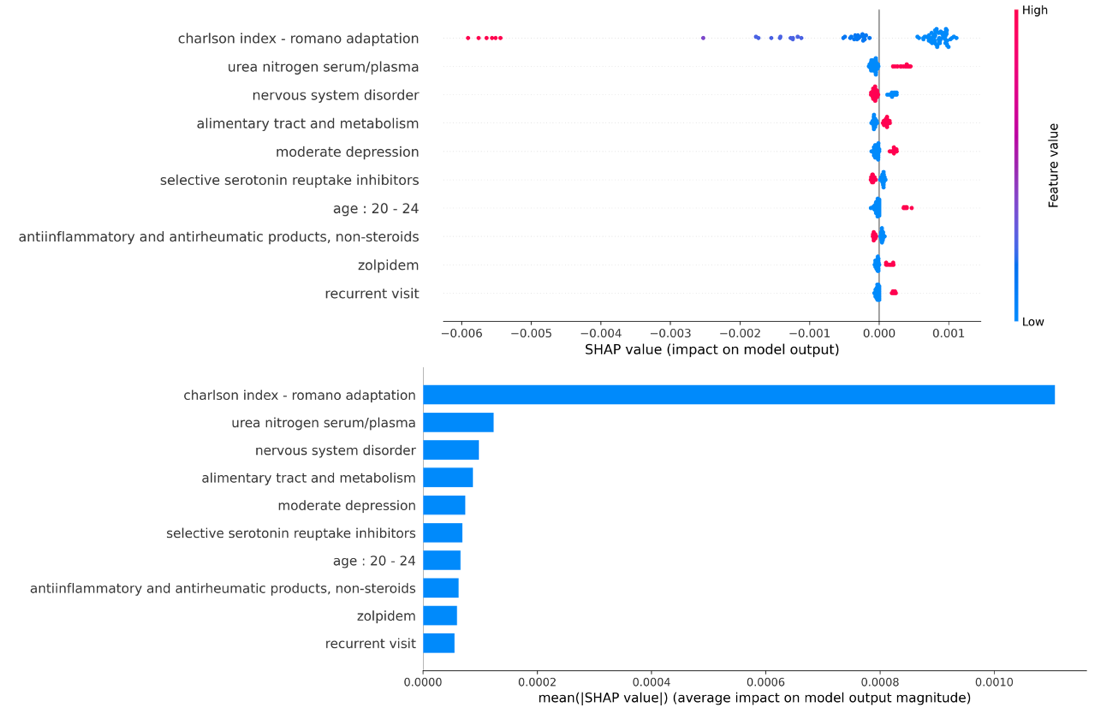


**Figure S4. SHAP plots of the local model in the Kyung Hee University Hospital (KHMC)**


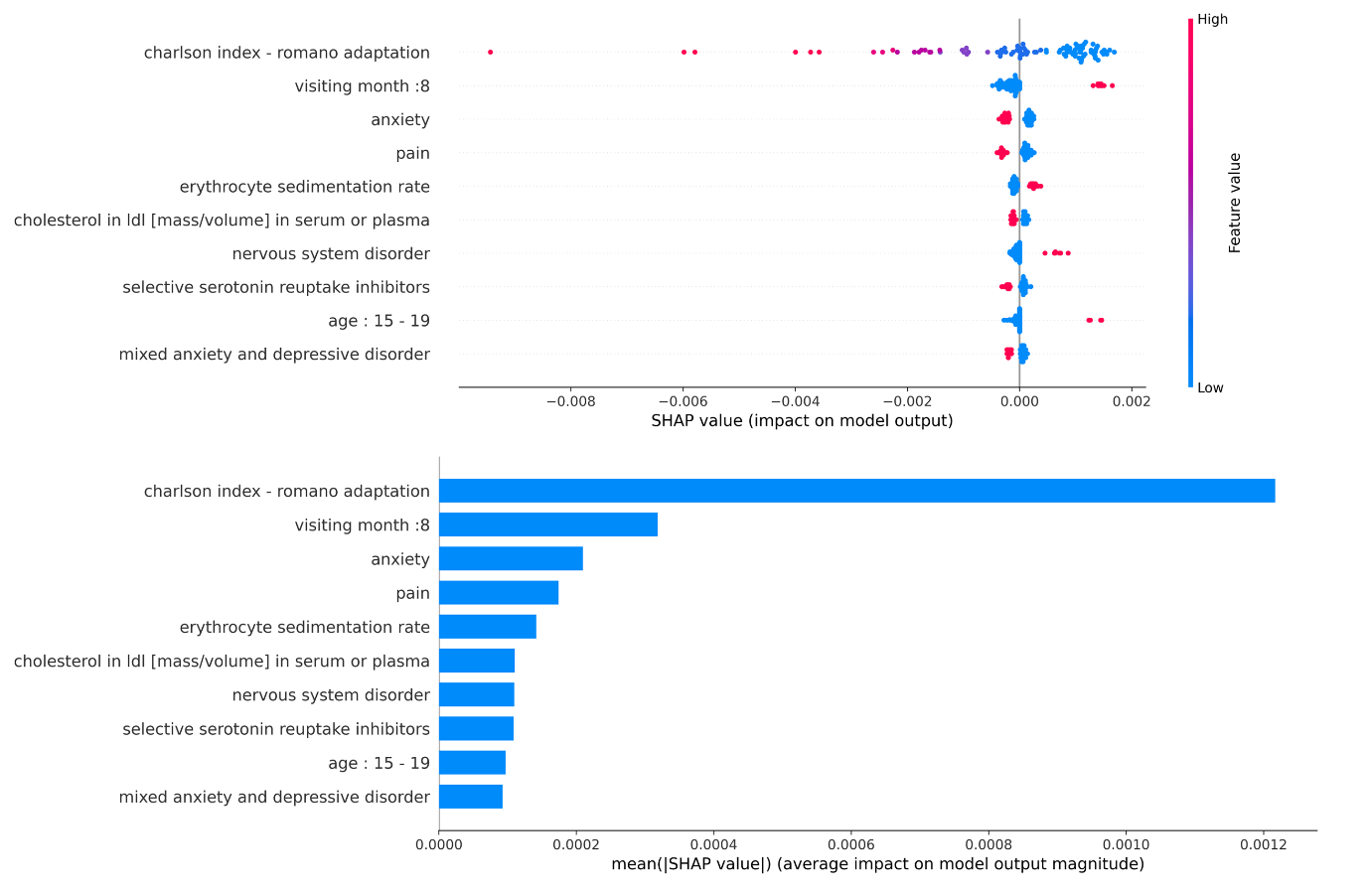


**Figure S5. SHAP plots of the local model in the Myongji Hospital (MJ)**


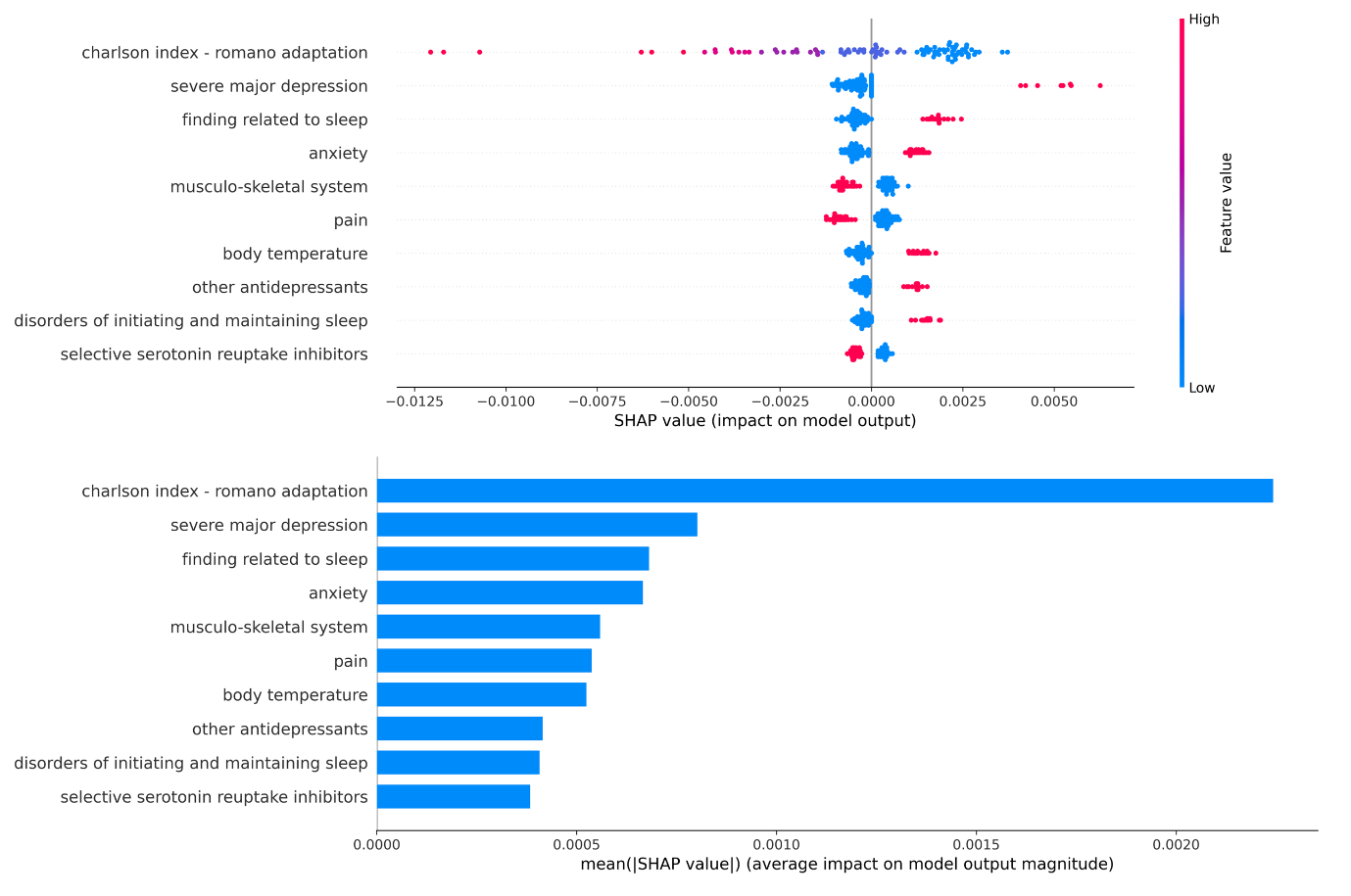


**Figure S6. SHAP plots of the local model in the Kangdong Sacred Heart Hospital (KDH)**


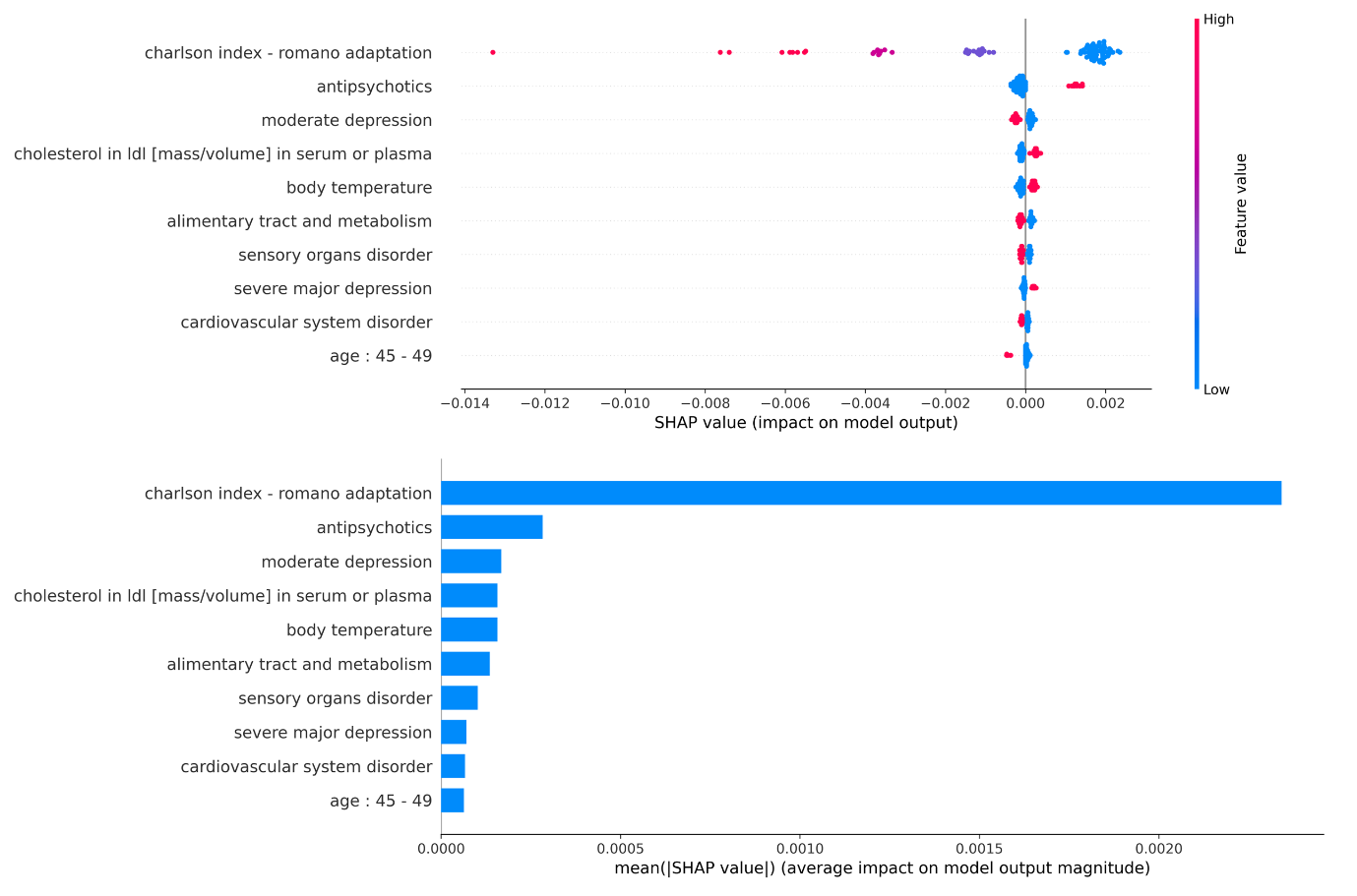

Supplement: Multimedia Appendix 1 [file jmir_v25i1e46165_app1.docx]
